# A systematic review and meta-analysis of acupuncture treatment for oral ulcer

**DOI:** 10.1097/MD.0000000000021314

**Published:** 2020-07-17

**Authors:** Hang Yan, Zhao Jin, Wenqin Jin, Yanmei Zhong, Huangping Ai, Yeke Wu, Qianrong Xu, Xiaoyan Bai, Donghao Liu, Wenhan Nie, Yuling Zuo

**Affiliations:** a Hospital of Chengdu University of Traditional Chinese Medicine; b Chengdu University of Traditional Chinese Medicine, Chengdu, Sichuan, P.R. China.

**Keywords:** acupuncture, oral ulcers, protocol, systematic review

## Abstract

**Background:**

Oral ulcers (OU) is a common oral mucosal disease manifested with obvious pain; in some studies, the efficacy of acupuncture in OU has been confirmed, but the systematic reviews and meta-analyses for them are lacking. Our aim is to evaluate the efficacy and safety of acupuncture in the treatment of OU.

**Methods:**

Relevant randomized controlled trials (RCTs), quasi RCTs and non-RCTs will be identified by systematic searching from the following electronic databases: PubMed, Embase, the Cochrane Library, Chinese Biomedical Literature Database, China National Knowledge Infrastructure, China Science and Technology Journal database, and Wanfang Data (since inception of the databases to present). In addition, ongoing trials will be retrieved from the Chinese Clinical Trial Register, World Health Organization International Clinical Trials Registry Platform, Clinical Trials, and The Clinical Trials Register. Grey literature will be also taken into consideration, including academic dissertation, minutes of the meeting from Chinese Biomedical Literature Database, China National Knowledge Infrastructure, China Science and Technology Journal database, and Wanfang Data. There are no language restrictions.

**Results:**

Ethical approval is not required because this study is based on published papers. After peer-review, the study will be disseminated in scientific journals and conferences.

**Conclusion:**

This systematic review will provide evidence for the efficacy and safety of acupuncture for Oral ulcers.

**Trial registration number:**

CRD42020144911.

## 1 Introduction

Oral ulcers (OU), the most frequent form of oral ulceration, characterized by recurrent oral mucosal ulceration in an otherwise healthy individual,^[1–3]^ occurs in the oral mucosa (lips, tongue, floor of the mouth, soft palate, uvula, etc) and pharyngeal mucosa.^[4]^ Among the general population, the incidence of OU is 5% to 20%.^[5–7]^ The primary lesion is neither a vesicle or a blister, but direct ulceration caused by epithelial necrosis exceeds the basement membrane, exposes nerve endings and causes discomfort or pain. Clinically, they present with painful ulcerations of different sizes, round or oval and clean edges.^[4,8]^ These lesions can hinder patients’ normal chewing, swallowing and speaking, and push patients to embrace frequent medical consultation.

The management of most of the ulcers is basically symptomatic; the basic aim is to relieve pain, short the healing time, diminish the size of the ulcer, decrease recurrence rate, and to increase disease-free intervals. Current treatment options available in management of OU include systemic and topical corticosteroids, antibiotics, multivitamins, adhesive pastes, local antiseptics, analgesics, anti-inflammatory, mouth rinses containing active enzymes, cauterization, and photobiomodulation therapy.^[9,10]^ Although there are various treatments, the curative effect is not stable and accompanied with lower risks of systemic adverse effects.^[11]^ Acupuncture involves the insertion of needles into specific anatomical points (termed acupoints) and has been used in eastern Asian countries for thousands of years. Several clinical experimental studies have indicated that acupuncture can facilitate ulcer healing of patients with OU.^[12–14]^ But the effect and safety of acupuncture for OU is still unclear; the results are controversial among different studies. Therefore, we designed this system evaluation scheme to evaluate the effect and safety of acupuncture for OU.

## 2 Methods

### 2.1 Design and registration of the review

The protocol has been registered on PROSPERO and registration number is CRD42020144911 and the protocol is based on the preferred reporting items for systematic reviews and meta-analyses protocols guidelines.^[15]^

### 2.2 Inclusion criteria for study selection

#### 2.2.1 Types of study

All the studies of acupuncture in the treatment of OU and the included studies will be all randomized controlled trials (RCTs). No limitation on language or publication types restriction. Nonrandomized clinical studies, quasi-RCTs, cluster RCTs, and case reports will be excluded.

#### 2.2.2 Types of participants

Trials will include participants who meet the diagnostic criteria of OU. All eligible study participants will be included in this review regardless of their age, race or gender. Trials will exclude study participants who are not appropriate to receive acupuncture therapy, such as pregnant or lactating women and those with additional severe diseases.

#### 2.2.3 Types of interventions

The patients of the experimental group should accept the acupuncture therapy that involves conventional acupuncture, electropuncture, ignipuncture, plum-blossom needle, and massaging acupoints. The efficiency of pharmacoacupuncture and acupoint injection will be analyzed by subgroup analysis, as their methods and theories are different from conventional acupuncture. There is no limitation on the duration and frequency of therapy.

#### 2.2.4 Types of outcome measures

##### 2.2.4.1 Primary outcomes

Primary outcome will be cure rate. We think that recovery means that the OU is completely healed.

##### 2.2.4.2 Secondary outcomes

Secondary outcome will be the rate of effectiveness. We think that effectiveness indicates rate of recurrence, healing time, and adverse events.

### 2.3 Data sources

Eights electronic databases and additional sources will be searched, including the Web of Science, PubMed, Cochrane Library, Embase, Chinese Biomedical Literature Database, Wanfang, China Science and Technology Journal database, China National Knowledge Infrastructure, and World Health Organization International Clinical Trials Registry Platform, Chinese Clinical Trial Register, Clinical Trials, Grey Literature Database, for potentially eligible studies. RCTs on acupuncture treatment in patients with postcholecystectomy syndrome will be searched independently by 2 reviewers in above mentioned resources.

### 2.4 Search strategy

The details will be adjusted according to the specific sources including Chinese Biomedical Literature Database, China National Knowledge Infrastructure, WanFang, China Science and Technology Journal database, Web of Science, Embase, PubMed, Cochrane Library, World Health Organization International Clinical Trials Registry Platform, Chinese Clinical Trial Register, Clinical Trials, and Grey Literature Database. The search strategy for PubMed is shown in Table 1.

**Table 1:**
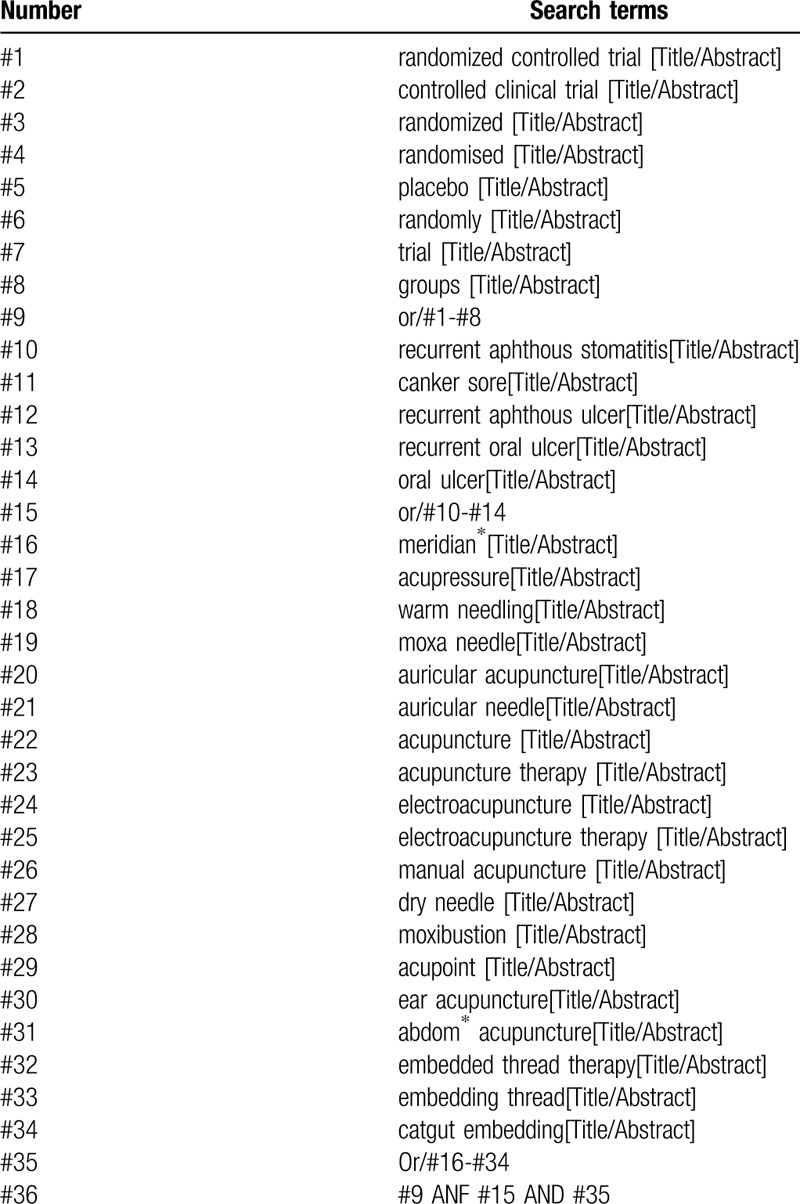
Search strategy used in Pubmed database.

### 2.5 Data collection and analysis

#### 2.5.1 Data management selection process and data items

For literature collection, the title and abstract of the literature will be read at first to eliminate duplicate literature and the eligible studies searched will be uploaded to a database set up through Endnote X8 and Excel software. Two review authors will make records independently through screening the titles, abstracts, and keywords. Data extraction will be conducted by 2 researchers independently through a standardized eligibility form. The general information of the selected articles will be extracted, including first author, year of publication, country, study design, sample size, detailed intervention, control treatment, duration of disease, duration of follow-up, and the like. When the data of articles are insufficient or ambiguous, one of the authors will contact the original author to request detailed about the research by E-mail or telephone or estimate the data.

**Figure 1:**
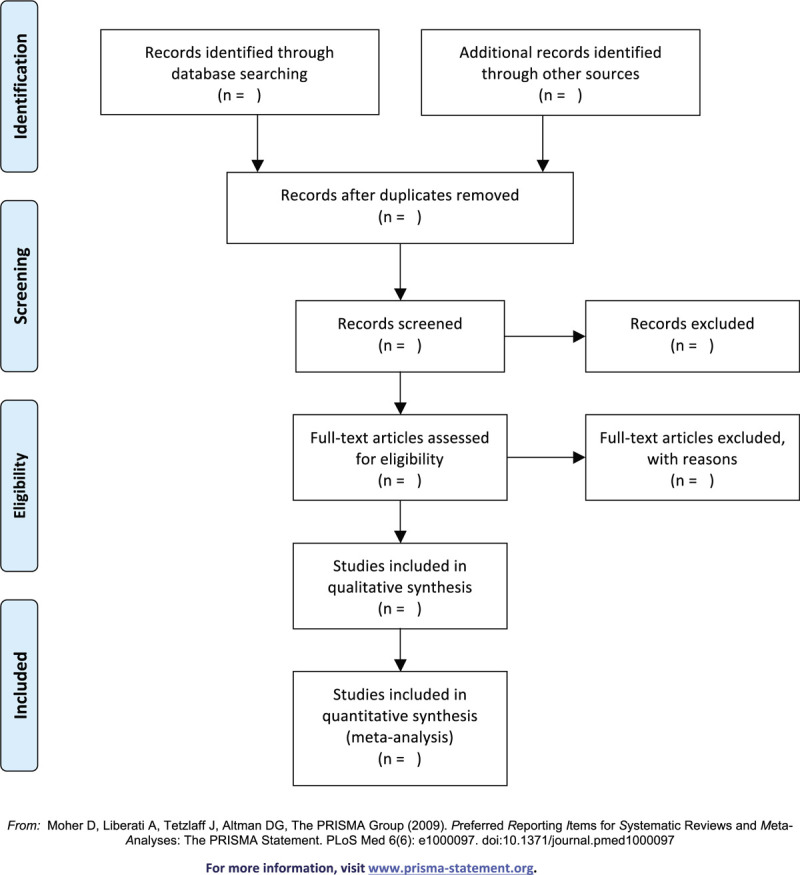
PRISMA flow diagram. PRISMA = preferred reporting items for systematic reviews and meta-analyses.

Any disagreement about the inclusion of the studies will be resolved through discussion between the 2 review authors. If the discussion cannot reach an agreement, the arbiter will make a final decision of the study selection. If authors are similar or incidence data are extracted from the same database, the study period will be assessed. Details of the selection procedure for studies are shown in a preferred reporting items for systematic reviews and meta-analyses protocols follow chart (Fig. 1).

#### 2.5.2 Assessment of risk of bias in included studies

At least 2 review author will independently assess the risk of bias of each included study in duplicate using the Cochrane risk of bias assessment tool. The assessed domains for each included study are: sequence generation, allocation concealment, blinding, completeness of outcome data, risk of selective outcome reporting, risk of other potential sources of bias. The risk level will be ranked as low risk of bias, unclear risk of bias, and high risk of bias. In case of disagreement, the arbiter shall be consulted to assist judgment.

#### 2.5.3 Dealing with missing data

If there is missing or incomplete data, we will contact the original investigator to verify the study characteristics and obtain missing numerical result data. If the missing data are not available, then this analysis will depend on the accessible data.

#### 2.5.4 Assessment of heterogeneity

According to the Cochrane Handbook, we will choose the *I*^2^ statistic to measure heterogeneity among the studies in every analysis. When *P* > .1, *I*^2^ < 50%, it is considered that there is no heterogeneity between the experiment, and the fixed effects model will be used for statistics, otherwise, the random effects model is adopted to analyze.

#### 2.5.5 Assessment of reporting biases

If the number of included studies is more than 10, funnel plots will be applied to measure publication bias. When funnel chart is evenly distributed, it can indicate no reporting bias, and vice versa.

#### 2.5.6 Data synthesis

The data will be analyzed and synthesized through Review Manager 5.3 which from the Cochrane Collaboration. The fixed effects model (*I*^2^ < 50%) or random effects model (*I*^2^ ≥ 50%) will be selected. All data will be analyzed with 95% confidence intervals. The dichotomous data will be analyzed by relative risk, and the continuous data will be analyzed by mean difference or standard mean difference.

#### 2.5.7 Subgroup analysis

If substantial heterogeneity is found, subgroup analysis will be implemented according to acupuncture types, outcome measures, and the like.

#### 2.5.8 Sensitivity analysis

Sensitivity analysis will be carried out to identify the quality and robustness of the results in the review. The principal criteria covers methodological quality, sample size, and analysis issue (such as missing data's efficacy). The meta-analysis will be operated repeatedly.

#### 2.5.9 Grading the quality of evidence

The quality of the research will be evaluated by utilizing the Grading of Recommendations Assessment, Development, and Evaluation approach.^[16]^ Utilizing the approach, the evidence will be classified as high, moderate, low, very low quality based on the risk of bias, inconsistency, indirectness, imprecision, and other domains. We assume that the quality of the evidence is the highest at first and gradually decreases according to the deficiencies of the study.

## 3 Discussion

OU as a mild and self-healing disease has a big impact on ordinary life of individuals because of recurrent and chronic oral mucosal ulcer. The objective of this systematic review is to evaluate the efficacy and safety of acupuncture for the treatment of OU.^[17,18]^

However, There may be some limitations in this systematic review, including different types of acupuncture, low quality articles, less strict methods, language limitations, and lack of research, which may lead to substantial heterogeneity. This is the first meta-analysis to assess the efficacy and safety of acupuncture for OU. We hope that our research will contribute to clinicians and public decision making.

## Author contributions

**Conceptualization:** Hang Yan, Zhao Jin, Yu-Ling Zuo.

**Data curation:** Hang Yan, Yanmei Zhong, Wen-Qin Jin.

**Formal analysis:** Yeke Wu, Qianrong Xu.

**Funding acquisition:** Yu-Ling Zuo.

**Methodology:** Huangping Ai, Wenhan Nie

**Project administration:** Yu-Ling Zuo.

**Supervision:** Xiaoyan Bai, Yanmei Zhong, Donghao Liu.

**Writing – original draft:** Hang Yan

**Writing – review & editing:** Hang Yan, Zhao Jin.
